# Removal of radioactive cesium from an aqueous solution *via* bioaccumulation by microalgae and magnetic separation

**DOI:** 10.1038/s41598-019-46586-x

**Published:** 2019-07-12

**Authors:** Ilgook Kim, Hee-Man Yang, Chan Woo Park, In-Ho Yoon, Bum-Kyoung Seo, Eun-Kyung Kim, Byung-Gon Ryu

**Affiliations:** 1 Decommissioning Technology Research Division, Korea Atomic Energy Research Institute (KAERI), Daejeon, 34057 Republic of Korea; 2 Advanced Biomass R&D Center, Korea Advanced Institute of Science and Technology (KAIST), 291, Daehak-ro, Yuseong-gu, Daejeon, Republic of Korea; 3 Microbial Research Department, Nakdonggang National Institute of Biological Resources (NNIBR), 137, Donam 2-gil, Sangju-si, 37242 Republic of Korea

**Keywords:** Biological techniques, Pollution remediation

## Abstract

We evaluated the potential sequestration of cesium (Cs^+^) by microalgae under heterotrophic growth conditions in an attempt to ultimately develop a system for treatment of radioactive wastewater. Thus, we examined the effects of initial Cs^+^ concentration (100–500 μM), pH (5–9), K^+^ and Na^+^ concentrations (0–20 mg/L), and different organic carbon sources (acetate, glycerol, glucose) on Cs^+^ removal. Our initial comparison of nine microalgae indicated that *Desmodesmus armatus* SCK had removed the most Cs^+^ under various environmental conditions. Addition of organic substrates significantly enhanced Cs^+^ uptake by *D*. *armatus*, even in the presence of a competitive cation (K^+^). We also applied magnetic nanoparticles coated with a cationic polymer (polyethylenimine) to separate ^137^Cs-containing microalgal biomass under a magnetic field. Our technique of combining bioaccumulation and magnetic separation successfully removed more than 90% of the radioactive ^137^Cs from an aqueous medium. These results clearly demonstrate that the method described here is a promising bioremediation technique for treatment of radioactive liquid waste.

## Introduction

The Fukushima Daiichi Nuclear Power Plant accident of 2011 released large amounts of radioactive nuclides into the environment. In particular, the release of radioactive cesium (^137^Cs) was a major concern because of its long half-life (30.2 years), high water solubility, and rapid uptake by terrestrial and aquatic organisms due to its chemical similarity to potassium (K^+^)^1,2^. This accident has thus led to the search for new methods that can prevent the adverse effects of pollution by radioactive nuclides, especially ^137^Cs.

Researchers have previously examined the effects of many chemical and biological techniques for removal of Cs^+^ and/or ^137^Cs from wastewater effluents. Biological technologies have attracted intense interest because they appear to be less expensive and more ecologically friendly than non-biological methods^3^. The uptake of radioactive compounds by microorganisms can be a metabolism-independent process (biosorption, a physiochemical process that does not require cellular energy) or a metabolism-dependent process (bioaccumulation, uptake into the cytoplasm by use of cellular energy). Previous research indicated that energy-independent processes play a minor role in Cs^+^ accumulation by microbes, because Cs^+^ is a very weak Lewis acid and only has limited interaction with ligands^4^. However, microorganisms can actively take up Cs^+^ *via* endogenous K^+^ transport systems^5^ because of the chemical similarity of Cs^+^ and K^+^. To the best of our knowledge, only a limited number of reports examined the use of microalgae for the bioaccumulation of environmental Cs^+^. Conventional separation techniques, such as chemical precipitation and ion exchange, are well-developed, but are expensive and inefficient when the environmental concentration of Cs^+^ is low. In general, contaminated environments have much lower concentrations of Cs^+^ than other co-occurring and competing cations. Thus, a biological method that uses microalgae, which can efficiently accumulate low levels of Cs^+^ in the presence of competing ions, is considered a promising approach^2^.

**Figure 1:**
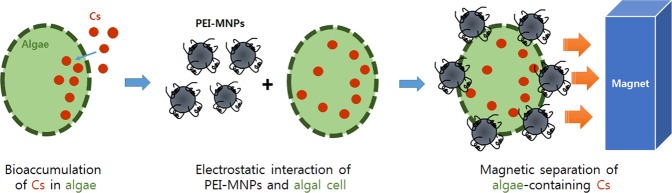
Overall process for removal of radioactive Cs *via* bioaccumulation by microalgae and magnetic separation.

In this study, we examined the feasibility of using microalgae with a magnetic separation system to remove Cs^+^ and ^137^Cs from aqueous solutions. Thus, we initially examined the Cs^+^-uptake capabilities of nine microalgae, including several newly isolated strains, under constant illumination to select the strain that best accumulates Cs^+^. We then selected the best of these nine microalgae and examined the effect of different environmental factors on Cs^+^ uptake and sequestration^6^, including pH, initial concentrations of different ions (Cs^+^, K^+^, and Na^+^), and different organic carbon sources (acetate, glycerol, and glucose). We also used polyethyleneimine (PEI)-coated magnetic nanoparticles (MNPs) to simultaneously recover the microalgae and ^137^Cs from solution. As a cationic surfactant, polyethlylenimine (PEI), which is known for its high density of positive charge, was introduced onto the surface of Fe_3_O_4_ nanoparticles to synthesize PEI-coated magnetic nanocomposites^7^. The proposed overall approach is summarized in Fig. 1.

## Results and Discussion

### Screening of Cs^+^-accumulating microalgae

**Figure 2:**
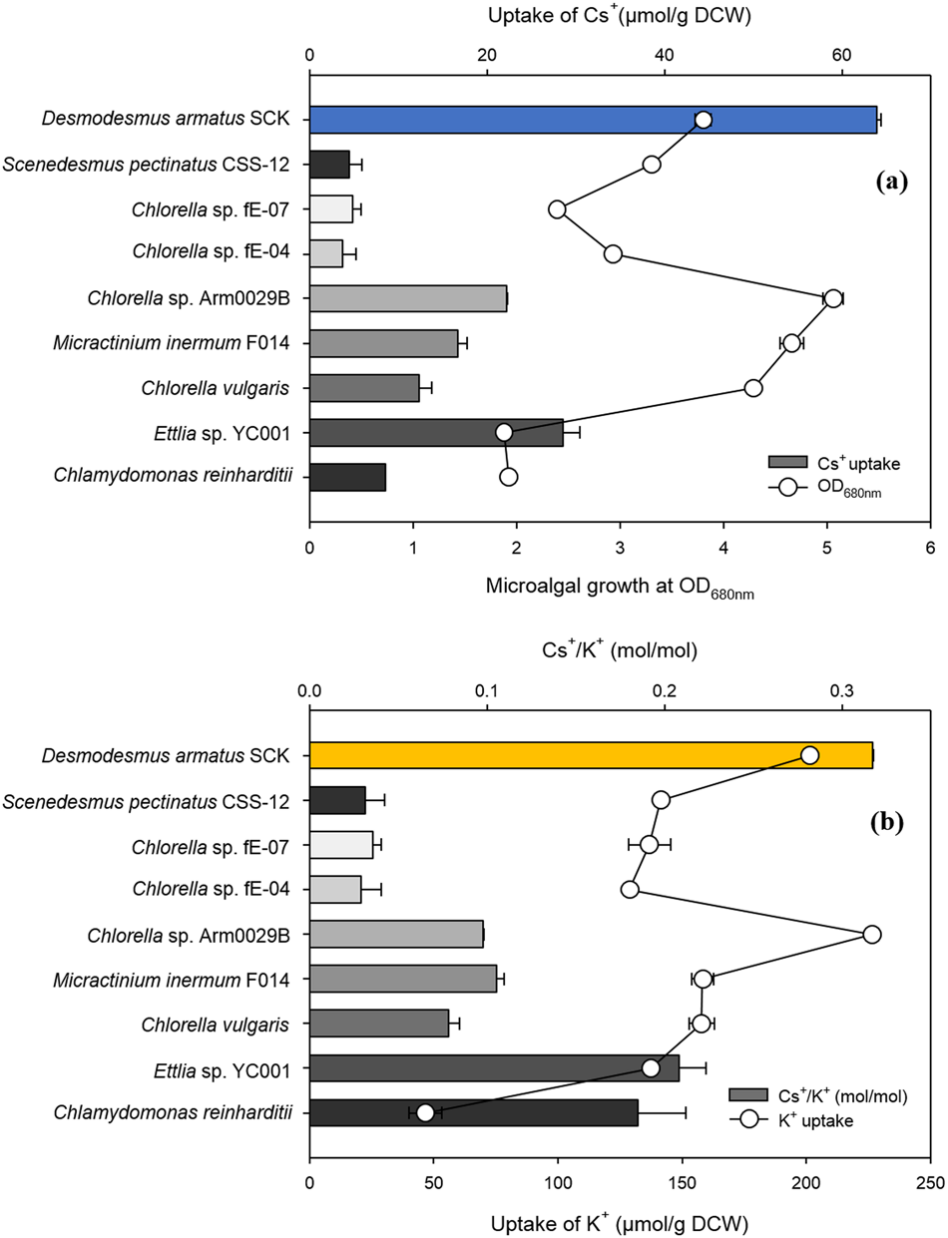
(**a**) Relationship of Cs^+^ uptake (bars) and algal growth (circles) and (**b**) relationship of the Cs^+^/K^+^ ratio (bars) and K^+^ accumulation (circles) by nine species of microalgae under constant illumination.

We initially grew the nine different microalgae under constant illumination in Cs^+^-containing TAP medium, following 3 days of growth in K^+^-starved TAP medium (Fig. 2a). Measurements of OD_680nm_ indicated *Chlorella* sp. Arm 0029B had the highest cell density, followed by *M*. *inermum* F014, *C*. *vulgaris*, and then *D*. *armatus* SCK. Measurements of Cs^+^ uptake by these microalgae indicated that *D*. *armatus* SCK removed the greatest amount of Cs^+^ (63.9 μmol/g DCW), followed by *Ettlia sp*. YC001 (28.6 μmol/g DCW), *Chlorella* sp. Arm 0029B (22.2 μmol/g DCW), and *M*. *inermum* F014 (16.7 μmol/g DCWM) (Fig. 2a). We also examined the cellular uptake of K^+^ from TAP medium without Cs^+^ (Fig. 2b). It is well known that cells take up Cs^+^ using their K^+^ transport systems, such as the K^+^/K^+^ and K^+^/H^+^ exchanger^5,8,9^.

However, our results indicated the uptake of K^+^ was not always proportional to uptake of Cs^+^. Based on uptake of K^+^, *D*. *armatus* SCK had the greatest Cs^+^ uptake at a Cs^+^/K^+^ molar ratio of 0.33. This indicated that uptake of Cs^+^ does not necessarily correlate with uptake of K^+^ or cell growth. Trans-membrane movement of monovalent cations like Cs generally occurs against a concentration gradient and thus energy is consumed to drive it. Additionally, it likely has to do with the plasma membrane-bound H^+^-ATPase, which acts to generate a transmembrane electrochemical proton gradient^4,5^. It is therefore possible that cation transports are coupled with H^+^ movements by either symport or antiport. Besides, the monovalent cation uptake may be mediated rather directly by K^+^-ATPase^4,5^. All this led to a mechanistic hypothesis that an apparent Cs^+^ uptake capacity is could be different for each cell type whose monovalent cation transport system has varied affinities toward Cs.

Previous studies reported that *Chlorella salina* and *Synechocystis* PCC 6803 accumulated Cs^+^ in the range of 0.8 to 0.49 nmol per 10^6^ cells when grown in BG-11 medium under constant illumination^10,11^. We found that *D*. *armatus* SCK sequestrated 2.08 nmol Cs^+^ per 10^6^ cells under our growth conditions, so this microalga appears to be a promising option for the removal of environmental Cs^+^. Thus, all of our subsequent experiments focused on this species.

### Effects of initial Cs^+^ concentration and pH on Cs^+^ removal

**Figure 3:**
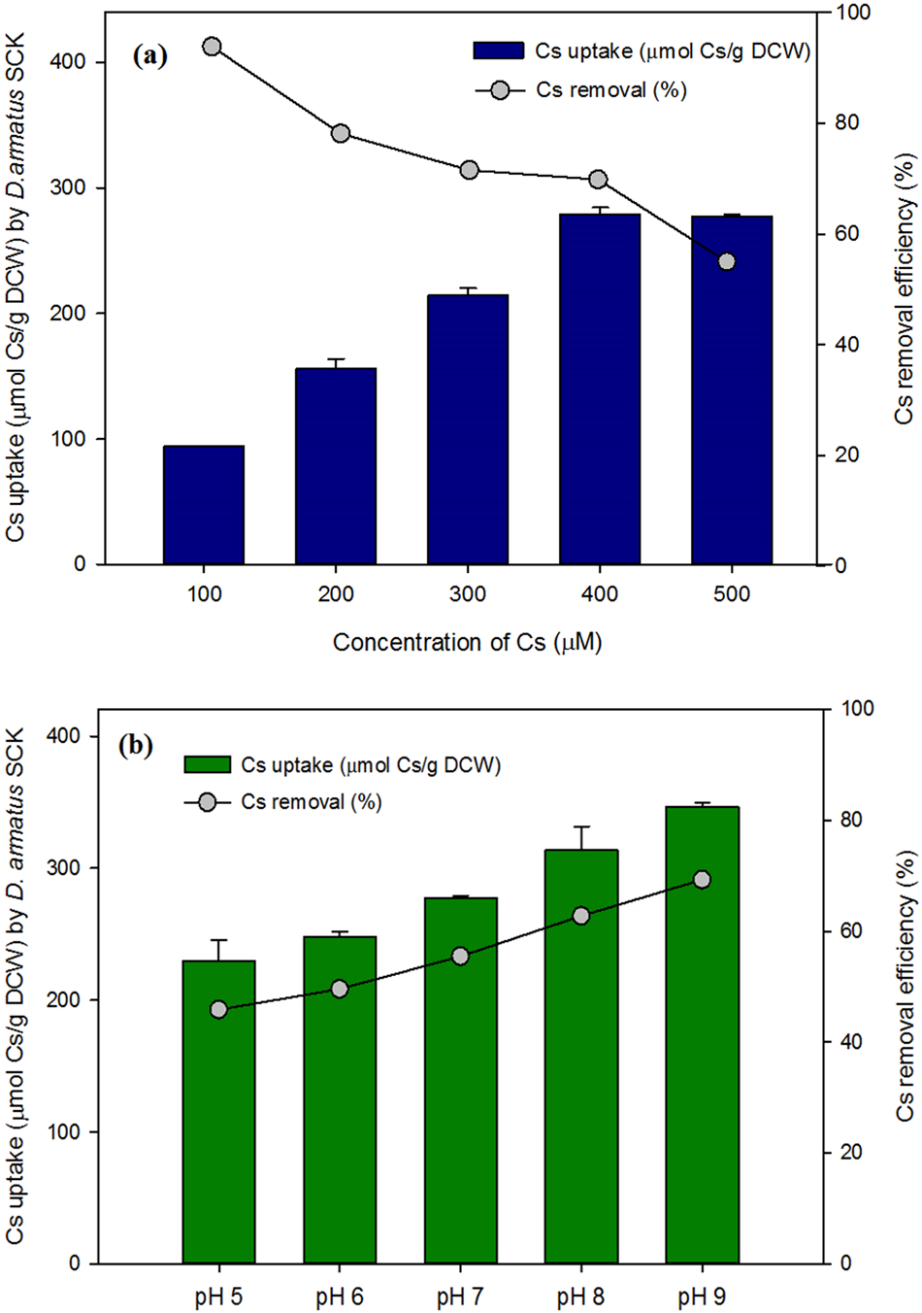
(**a**) Effect of initial Cs^+^ concentration on Cs^+^ uptake (bars) and removal efficiency (circles) at pH 7, and (**b**) effect of pH on Cs^+^ uptake (bars) and removal efficiency (circles) at 400 μM Cs^+^ by *D*. *armatus* SCK cells grown in TAP medium.

Cs^+^ is a hard metal that is generally non-toxic to microorganisms because of its weak coordinating ability. Moreover, several hard metals are essential nutrients for microbial growth, and so they are readily accumulated^4^. Thus, we examined the effect of the initial Cs^+^ concentration and pH on Cs^+^ uptake by *D*. *armatus* SCK (Fig. 3). The results show that Cs^+^ uptake increased with an increasing initial Cs^+^ concentration, with an apparent saturation near 400 to 500 μM. The maximum equilibrium uptake of Cs^+^ was 280 μmol/g DCW and the removal efficiency was 70% at 400 μM. Tomioka *et al*.^12^ reported that several strains of *Rhodococcus* accumulated Cs^+^ in the range of 98.3 to 395 μmol/g cells weight for initial Cs^+^ concentrations of 0.01 to 1 mM, but that the Cs^+^ uptake did not increase when the extracellular Cs^+^ concentration was greater than 100 μM^12^.

The pH of the growth medium can impact the bioaccumulation or adsorption of Cs^+^. Our results show that the bioaccumulation of Cs^+^ by *D*. *armatus* SCK was efficient at pH values between 5 and 9, and the greatest uptake and removal efficiency were at pH 9. This result is similar to those reported for cyanobacterial removal of Cs^+^. In particular, optimal Cs^+^ accumulation by *Synechocystis* PCC 6803 and *Rhodococcus* strain occurred under alkaline conditions (pH 9)^1,10^, probably because plasma membrane depolarization at low pH inhibits Cs^+^ uptake^10^.

### Effects of K^+^ and Na^+^ on Cs^+^ removal

**Figure 4:**
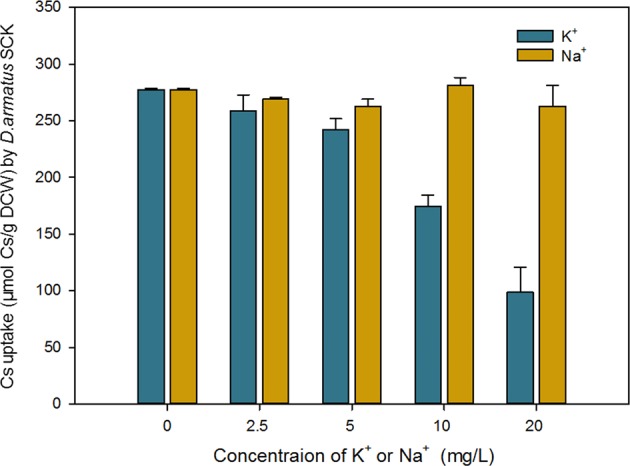
Effect of K^+^ and Na^+^ concentration on Cs^+^ uptake by *D*. *armatus* SCK grown in TAP medium containing 400 μM Cs^+^.

Cs^+^ is an alkali monovalent cation that cells can transport because of its similarity to K^+^. Thus, we examined the effects of K^+^ and Na^+^ concentration on Cs^+^ uptake by *D*. *armatus* SCK (Fig. 4). The results show that the Na^+^ concentration had little or no effect on Cs^+^ uptake in the concentration range tested, but that Cs^+^ uptake declined as the K^+^ concentration was above 10 mg/L. Similar to other microbes, our results show that Cs^+^ accumulation may occur through sharing K^+^-transport channel, not Na^+^-migration route^5^. Similarly, Cs^+^ accumulation by *Synechocystis* sp. Strain PCC 6803 and *C*. *emersonii* declined as the concentration of K^+^ increased^5,10^. These results also suggest that the K^+^-transport system(s) of phototrophic microalgae, which are not specific to K^+^, play an important role in accumulation of Cs^+^^12^. The K^+^ concentrations in natural fresh water and ground water systems typically range from 0.5 to 3 mg/L, levels that have no apparent impact on Cs^+^ uptake by *D*. *armatus* SCK^2^. However, our results indicate that when this strain is used in laboratory or bioengineering studies, the K^+^ concentration should be 10 mg/L or less.

### Effects of different organic carbon sources on Cs^+^ removal

**Figure 5:**
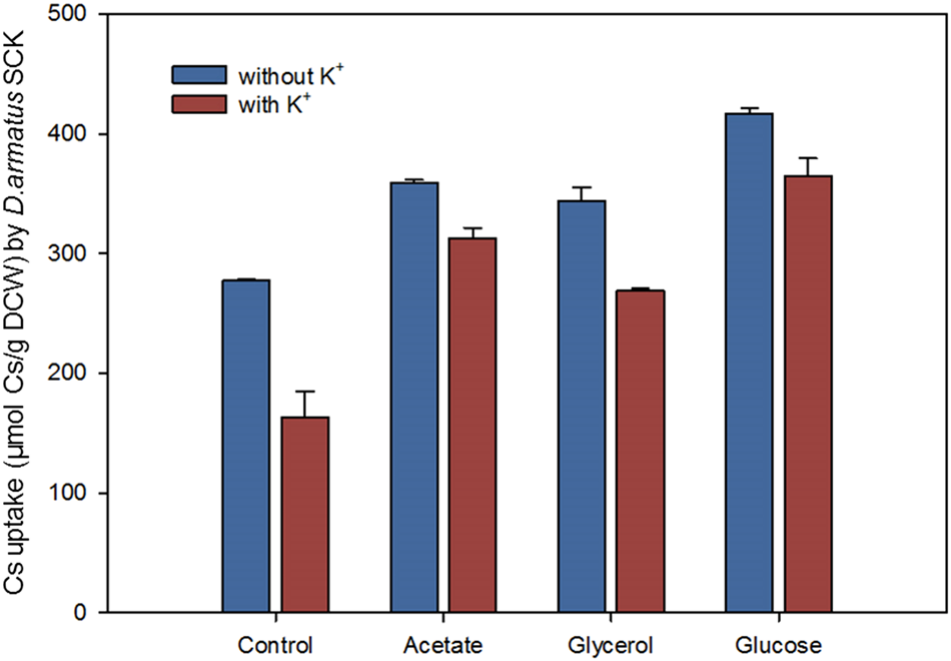
Effect of 500 μM K^+^ and three different carbon sources on uptake of Cs^+^ by *D*. *armatus* SCK when grown in liquid medium containing 400 μM Cs^+^.

We investigated the effects of three organic carbon sources (acetate, glycerol, and glucose; concentration: 1 g-Carbon/L) on Cs^+^ uptake by *D*. *armatus* SCK with or without 500 μM K^+^ (Fig. 5). The results show that each carbon source notably increased Cs^+^ uptake in the presence or absence of K^+^, and that glucose had the greatest effect. In agreement, a previous study also reported increased net K^+^ uptake by heterotrophic *Rhodococcus* cells when glucose was added to the growth medium^13^.

Glucose-induced net uptake of K^+^ and Cs^+^ is likely due to stimulation of an electro-neutral ATP-dependent K^+^/H^+^ exchange, because glucose metabolism acidifies the cell interior. Ohunki *et al*.^14^ also found that glucose stimulated the accumulation of Cs^+^ in a fungal strain^14^. Our results also indicate that glucose and other carbon sources can act as energy sources that increase Cs^+^ accumulation by microalgae under heterotrophic conditions. In contrast, other research indicated that *Chlorella* accumulated 2-fold less Cs^+^ in chemoheterotrophic conditions relative to photoautotrophic conditions^5^. Few previous studies have examined the effects of other organic substrates, such as acetate and glycerol, on the uptake of Cs^+^ by phototrophic microalgae. Our results (Fig. 5) indicate that acetate and glycerol significantly increased the uptake of Cs^+^, although glucose had a stronger effect. Acetate is a volatile fatty acid that microalgae can directly convert into acetyl-CoA (an intermediate in the synthesis of cellular fatty acids) *via* the pyruvate pathway in the absence of glucose^15^, so this may explain its effect (Fig. 5). Although glycerol was not as effective as glucose, it can be considered as an alternative to improve the accumulation of Cs^+^ by *D*. *armatus* SCK. Therefore, from an economic perspective, these two low-cost organic carbon sources (acetate and glycerol) have potential for enhancing the accumulation of Cs^+^ during the heterotrophic growth of *D*. *armatus* SCK.

### Removal of ^137^Cs using bioaccumulation and magnetic separation

**Figure 6:**
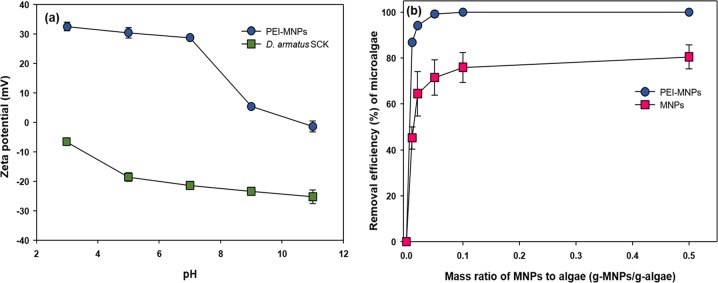
(**a**) Zeta potentials of PEI-MNPs and *D*. *armatus* SCK at different pHs, (**b**) Recovery efficiency of *D*. *armatus* SCK according to the mass ratio of MNPs to algae.

**Figure 7:**
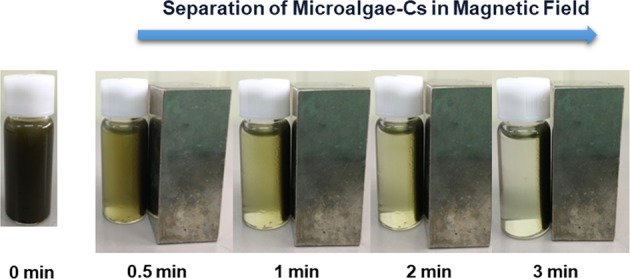
Time course of separation of Cs^+^-containing microalgae using PEI-MNPs in a magnetic field.

We measured the separation of microalgae containing Cs^+^ from liquid medium by use of PEI-MNPs. In an aqueous solution, the surface of metal oxide nanoparticles contains hydroxyl groups, which undergo pH-dependent protonation/deprotonation. Fe_3_O_4_ nanoparticles are generally negatively charged at pH 7^16,17^. Therefore, the negative surface potential of algal cells (*D*. *armatus* SCK: −21.4 ± 0.92 mV at pH 7) makes them strongly attracted to Fe_3_O_4_ nanoparticles that are coated with PEI, which have a high-density cationic charge (+28.7 ± 0.64 mV at pH 7) (Fig. 6a). As reported in previous studies, the addition of cationic functional groups, such as PEI, to the surface of coated particles increases the effectiveness of separation^18–20^. As shown in Fig. 6b, the maximum recovery efficiency (~100%) was achieved when the mass ratio exceeds 0.05 g-PEI-MNPs/g-algae, which showed the improved magnetic separation than that of naked Fe_3_O_4_ particles. We also found that the complex of magnetic nanocomposites and microalgal cells were easily separated within 3 min in a magnetic field (Fig. 7). Thus, this magnetic harvesting method has potential for the efficient separation of microalgae containing ^137^Cs because it is simple, rapid, and consumes very little energy.

**Figure 8:**
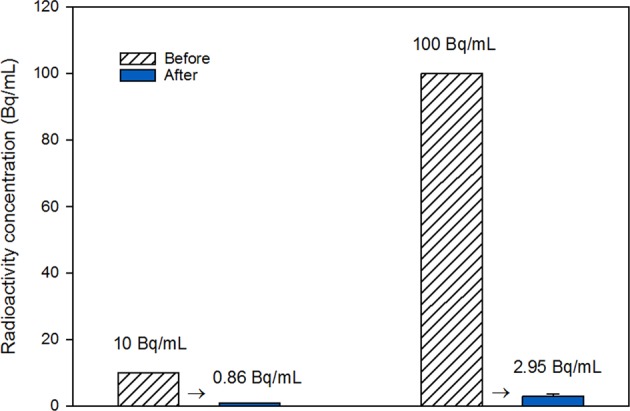
Change of ^137^Cs concentration in the liquid medium following bioaccumulation by *D*. *armatus* SCK and magnetic separation.

We also examined the use of *D*. *armatus* SCK cells followed by magnetic separation for removal of ^137^Cs from a medium with glucose (1 g/L) and 10 Bq/mL or 100 Bq/mL of ^137^Cs (Fig. 8). Measurement of radioactivity in the liquid medium before and after treatment indicated these cells sequestered 91.4% and 97.1% of ^137^Cs at 10 and 100 Bq/mL of ^137^Cs, respectively (Fig. 8). These data are meaningful because low levels of ^137^Cs are often difficult to remove using conventional methods in aqueous systems.

## Materials and Methods

### Screening of Cs-accumulating microalgae

Four microalgae that accumulate Cs^+^ were isolated from a local sewage treatment plant (*Desmodesmus armatus* SCK) and a lake (*Scenedesmus pectinatus* CSS-12, *Chlorella* sp. fE-04, and *Chlorella* sp. fE-07) in Daejeon, Republic of Korea. An additional five microalgae from the culture collections of the Korea Research Institute of Bioscience and Biotechnology (KRIBB) in Dajeon, Korea (*Chlorella* sp. ArM0029B, *Ettlia* sp. YC001, and *Chlamydomonas reinhardtii* CC124), Pusan National University in Pusan, Korea (*Micractinium inermum* F014), and University of Texas (UTEX), Austin, TX, USA (*Chlorella vulgaris* UTEX265). For screening of Cs-accumulating microalgae, each strain was grown in 1 L vessel in Tris-acetate phosphate (TAP) medium (pH = 7) with 100-μmol/L of CsCl and the following additional components: 25 mL of TAP salts, 0.375 mL of phosphate solution, 1 mL of Hunter’s trace elements, 1 mL of glacial acetic acid, and 2.42 g of Tris^15^. The initial cell concentration was adjusted to an optical density of 0.2 at 680 nm. These algal strains were cultivated for 7 days in baffled hybrid flasks on a shaker (120 rpm) at 25 °C.

### Effects of various parameters on Cs removal by microalgae

To evaluate the maximum uptake capability of Cs^+^ by microalgae, cultivated cells were transferred to K^+^-depleted TAP medium and maintained for 3 days (Supplementary Fig. S1). Then, cells in the early stationary growth phase were collected by centrifugation at 7000 rpm for 10 min, and the bio-pellets were suspended in a 20-mM Tris buffer solution containing CsCl, with the biomass adjusted to 1 g/L dry cell weight (DCW). Cell suspensions were then incubated at 25 °C for 24 h with rotary shaking (120 rpm) under continuous illumination. The effects of the initial concentrations of Cs^+^, K^+^ and Na^+^, and various organic carbon sources (acetate, glycerol, and glucose) were examined at 25 °C under continuous illumination. The removal efficiency of non-radioactive Cs^+^ was calculated as:

1 $${\rm{Removal}}\,{\rm{efficiency}}\,( \% )=({C}_{i}-{C}_{f})/{C}_{i}\times 100$$

where *C*_*i*_ and *C*_*f*_ are the initial (i) and final (f) concentrations of Cs^+^.

### Synthesis of PEI-Fe_3_O_4_ nanoparticles and magnetic separation

PEI (polyethyleneimine)-coated magnetic nanoparticles (MNPs) were synthesized according to a previously reported procedure^7^. First, iron salts (0.99 g FeCl_2_∙4H_2_O and 2.7 g FeCl_3_∙6H_2_O) were dissolved in 100 mL of deionized water, and deoxygenated with nitrogen gas at 80 °C. Subsequently, 10 mL of NH_4_OH (25% by wt.) was added and stirred for 0.5 h. After cooling to room temperature, the precipitated nanoparticles were separated with a magnet and washed four times with deionized water. Then, the Fe_3_O_4_ nanoparticles were added to a PEI solution (MW = 2 kDa) in phosphate buffer at pH 7.3 (10% by vol.). Finally, the PEI-MNPs were collected using a magnetic field and washed three times with deionized water. After synthesis of the nanoparticles, the zeta potentials of PEI-MNPs and prepared microalgae were measured by a Zetasizer instrument (Nano-ZS, Malvern, UK).

For the magnetic separation and removal of radioactive ^137^Cs, *D*. *armatus* SCK cells were cultivated in a Tris buffer solution containing 10 or 100 Bq/mL of ^137^Cs, and the PEI-MNPs were mixed with the cultures for 1 min. Then, the microalga-nanoparticle aggregates containing ^137^Cs were separated from the medium using an external permanent magnet within 3 min. After separation, the radioactivity concentration of the solution was measured to calculate the removal efficiency, as described above.

### Analytical methods

To quantify cell growth, the optical density of each sample was measured at 680 nm using a UV-visible spectrophotometer (UV-1800; Shimadzu, Japan). Cell counts were performed using an optical microscope (DM2500; Leica, Switzerland) with a hemocytometer.

After the Cs removal experiments, the supernatant was filtered through a PVDF membrane filter (0.2 μm), and the amount of non-radioactive Cs^+^ remaining in the filtrate was quantified using inductively coupled plasma-mass spectrometry (ICP-MS; ELAN DRC II, Perkin-Elmer). Thus, the cellular uptake of Cs^+^ was determined by measuring the change in Cs^+^ concentration of the growth medium. The levels of K^+^ and Na^+^ were determined by ion chromatography (883 Basic IC plus; Metrohm AG, Switzerland) using an anionic column (Metrosep A Supp 5–150/4.0; Metrohm AG, Switzerland). The ^137^Cs concentration was determined using γ-spectrometry (Canberra, Genie 2000).

## Conclusion

The aim of this work was to evaluate the ability of microalgae to remove Cs^+^ and ^137^Cs from aqueous solutions. Initial experiments indicated that a novel strain, *D*. *armatus* SCK, was the most effective of nine tested strains in the removal of Cs^+^. Our results also showed that *D*. *armatus* SCK accumulated high levels of Cs^+^ in the presence of competitive cations (Na^+^ and K^+^), that acetate and glycerol (inexpensive carbon sources) enhanced the uptake of Cs^+^, and that uptake was greater at a higher pH. Use of *D*. *armatus* SCK for bioaccumulation and PEI-MNPs for magnetic separation of cells led to highly effective removal of ^137^Cs from aqueous solutions. The use of microalgae-magnetic particles with an inexpensive organic substrate appears to have great potential for bioremediation of ^137^Cs-polluted environments.

## Electronic supplementary material

Supplementary Information
