# Supplementary material for: Removal of radioactive cesium from an aqueous solution via bioaccumulation by microalgae and magnetic separation
Source: Sci Rep. 2019 Jul 12;9:10149. doi: 10.1038/s41598-019-46586-x (PMC6626050; doi:10.1038/s41598-019-46586-x)

***Supplementary Information***

**Removal of radioactive cesium from an aqueous solution *via* bioaccumulation by microalgae and magnetic separation**

# Ilgook Kim1, Hee-Man Yang1, Chan Woo Park1, In-Ho Yoon1, Bum-Kyoung Seo1, Eun Kyung Kim^2^, and Byung-Gon Ryu3,*

^1^ Decommissioning Technology Research Division, Korea Atomic Energy Research Institute (KAERI), Daejeon, 34057, Republic of Korea

2 Advanced Biomass R&D Center, Korea Advanced Institute of Science and Technology (KAIST), 291, Yuseong-gu, Daejeon 305-701, Republic of Korea

3 Microbial Research Department, Nakdonggang National Institute of Biological Resources (NNIBR), 137, Donam 2-gil, Sangju-si 37242, Republic of Korea

# *Corresponding authors:

# Byung-Gon Ryu

# Tel: +82 54 530 0873; Fax: +82 54 350 0879

# E-mail address: tesia@nnibr.re.kr

**Figure S1**. Effect of presence of the K^+^ in the medium on Cs^+^ removal efficiency by microalgae (TAP-25: typical TAP medium containing K^+^ at 25°C; TAP-K-25: typical TAP medium except for K^+^ at 25°C). Conditions: pH = 7.0, operating period = 24 h, initial concentration of Cs^+^ = 100 μmol/L, working volume = 250 mL, stirring speed = 120 rpm, light intensity = 120 μmol/m^2^/s.


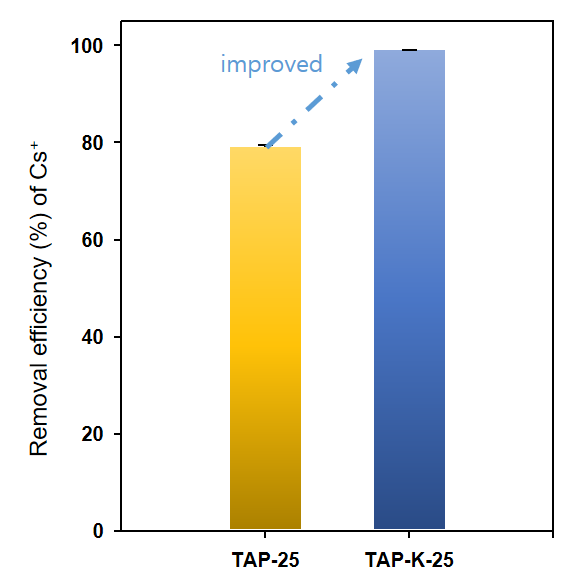

Supplement: Supplementary file 1 — Supplementary Information [file 41598_2019_46586_MOESM1_ESM.docx]
